# Incremental Diagnostic Value of Zero-Echo-Time Magnetic Resonance Imaging for Osseous Cervical Neural Foraminal Stenosis: A Retrospective Multireader Study

**DOI:** 10.3390/diagnostics16213597

**Published:** 2026-10-31

**Authors:** Jiandong Guo, Shasha Zeng, Wenke Tang, Tiejun Zhang, Shilong Zhuang, Jichao Zhu, Ping Xiao, Yafen Li, Wenxin Li, Song Xu, Gan Cao

**Affiliations:** 1 Department of Radiology, Shenzhen Hospital (Futian) of Guangzhou University of Chinese Medicine, 6001 Beihuan Avenue, Futian District, Shenzhen 518000, China; gjdshenzhen@21cn.com (J.G.); zss13632752184@163.com (S.Z.); 15136715116@163.com (W.T.); zhangtiejun999@163.com (T.Z.); 18819005552@163.com (S.Z.); 2 Laboratory of Molecular Imaging and Medical Intelligence, Department of Radiology, Longgang Central Hospital of Shenzhen; Shenzhen Clinical Medical College, Guangzhou University of Chinese Medicine; Longgang Clinical Institute of Shantou University Medical College, 6082 Longgang Avenue, Longgang District, Shenzhen 518116, China; jackeyzjc@163.com (J.Z.); xiaoping0925@163.com (P.X.); 3 School of Health Science and Engineering, University of Shanghai for Science and Technology, 334 Jungong Road, Yangpu District, Shanghai 200093, China; liyafen@foxmail.com (Y.L.); 18721323397@163.com (W.L.)

**Keywords:** cervical spine, neural foraminal stenosis, zero-echo-time imaging, magnetic resonance imaging, computed tomography, diagnostic accuracy

## Abstract

**Background/Objectives:** Conventional cervical magnetic resonance imaging (MRI) depicts cortical bone poorly, whereas computed tomography (CT) adds radiation exposure. We evaluated whether adding zero-echo-time (ZTE) imaging improves detection of CT-defined grade ≥2 foraminal stenosis. **Methods:** This single-centre retrospective diagnostic accuracy study included 81 consecutive eligible patients evaluated between October 2024 and June 2025 who underwent cervical MRI and CT within 14 days. Two blinded musculoskeletal radiologists graded 810 foramina from C2-C3 through C6-C7 using conventional MRI and MRI + ZTE. A study-specific scale incorporated narrowing, nerve-root morphology, and surrounding fat appearances. Consensus CT grading was the reference standard; grade ≥2 defined a positive foramen. Paired differences were assessed with patient-clustered bootstrap resampling. **Results:** Mean age was 48.9 ± 13.6 years; 55 patients were women. CT identified grade ≥2 stenosis in 150/810 foramina (18.5%). Adding ZTE increased sensitivity from 68.7% to 87.3% for Reader 1 and from 17.3% to 35.3% for Reader 2. Sensitivity differences were 18.7 percentage points (95% confidence interval [CI], 10.6–27.1) and 18.0 percentage points (95% CI, 6.6–30.3), respectively. Specificity increased from 90.5% to 96.8% and from 96.7% to 97.6%, respectively. Areas under the receiver operating characteristic curve increased by 0.115 (95% CI, 0.076–0.164) and 0.121 (95% CI, 0.061–0.184). Binary interreader agreement increased numerically from κ = 0.25 to κ = 0.41. **Conclusions:** Adding ZTE improved detection of CT-defined grade ≥2 foraminal stenosis, although sensitivity remained low for one reader. Effects on CT use, management decisions, and clinical outcomes require prospective evaluation.

## 1. Introduction

Cervical radiculopathy commonly results from disc disease and osseous foraminal narrowing, including uncovertebral and facet osteophytes [1]. Accurate assessment of foraminal stenosis informs conservative treatment, image-guided interventions, and surgical decompression [1,2]. Magnetic resonance imaging (MRI) is generally the first-line modality because it depicts neural and soft-tissue structures without ionising radiation [1]. Conventional T1- and T2-weighted sequences depict cortical bone and osteophytes less clearly. The short T2/T2* relaxation times of cortical bone produce signal voids, which may reduce confidence in grading osseous narrowing [3,4].

Computed tomography (CT) depicts cortical bone with high spatial resolution and is widely used to characterise bony foraminal stenosis [1,5]; however, CT adds cost, examination time, and ionising radiation exposure [4,6]. MRI techniques that provide bone contrast without radiation are therefore of interest [5,7]. Zero-echo-time (ZTE) MRI and ultrashort echo time (UTE) imaging capture signals from short-T2 tissues and can produce CT-like bone contrast [3,4]. These techniques have previously been assessed for spinal bone visualisation and cervical foraminal stenosis [8,9,10].

The incremental value of adding ZTE to conventional cervical MRI for detecting CT-defined moderate-to-severe foraminal stenosis remains incompletely characterised across readers. Recent cervical bone-MRI studies have evaluated osseous contributions to narrowing, diagnostic confidence, and reconstruction effects on image quality and pathology assessment [9,11,12]. Foraminal grading systems differ in their definitions and imaging planes, limiting comparisons across studies [13]. A paired CT-referenced comparison may help define the value of ZTE as an add-on sequence for suspected cervical radiculopathy.

We compared conventional MRI alone with MRI + ZTE for detection of CT-defined grade ≥ 2 cervical foraminal stenosis in a retrospective multireader study. We hypothesised that adding ZTE would improve detection and increase interreader agreement. Secondary objectives were to evaluate ZTE image quality and ZTE–CT morphometric agreement.

## 2. Materials and Methods

### 2.1. Study Design and Patient Population

This single-centre retrospective diagnostic accuracy study was approved by the institutional medical ethics committee on 15 January 2025, with a waiver of written informed consent. Reporting followed the Standards for Reporting Diagnostic Accuracy Studies 2015 and the Strengthening the Reporting of Observational Studies in Epidemiology recommendations, as applicable [14,15].

All MRI and CT examinations were obtained during routine clinical care; no patient was prospectively recruited or scheduled for research imaging. The retrospective dataset was assembled, and images were interpreted after ethics approval. The study followed the Declaration of Helsinki [16].

Patients underwent multidetector CT and 3.0-T cervical MRI, including ZTE. The index tests were conventional MRI alone and MRI + ZTE, evaluated in separate blinded sessions. The target classification was moderate-to-severe foraminal stenosis, defined as consensus CT grade 2 or 3 on the study-specific composite scale described below.

**Figure 1:**
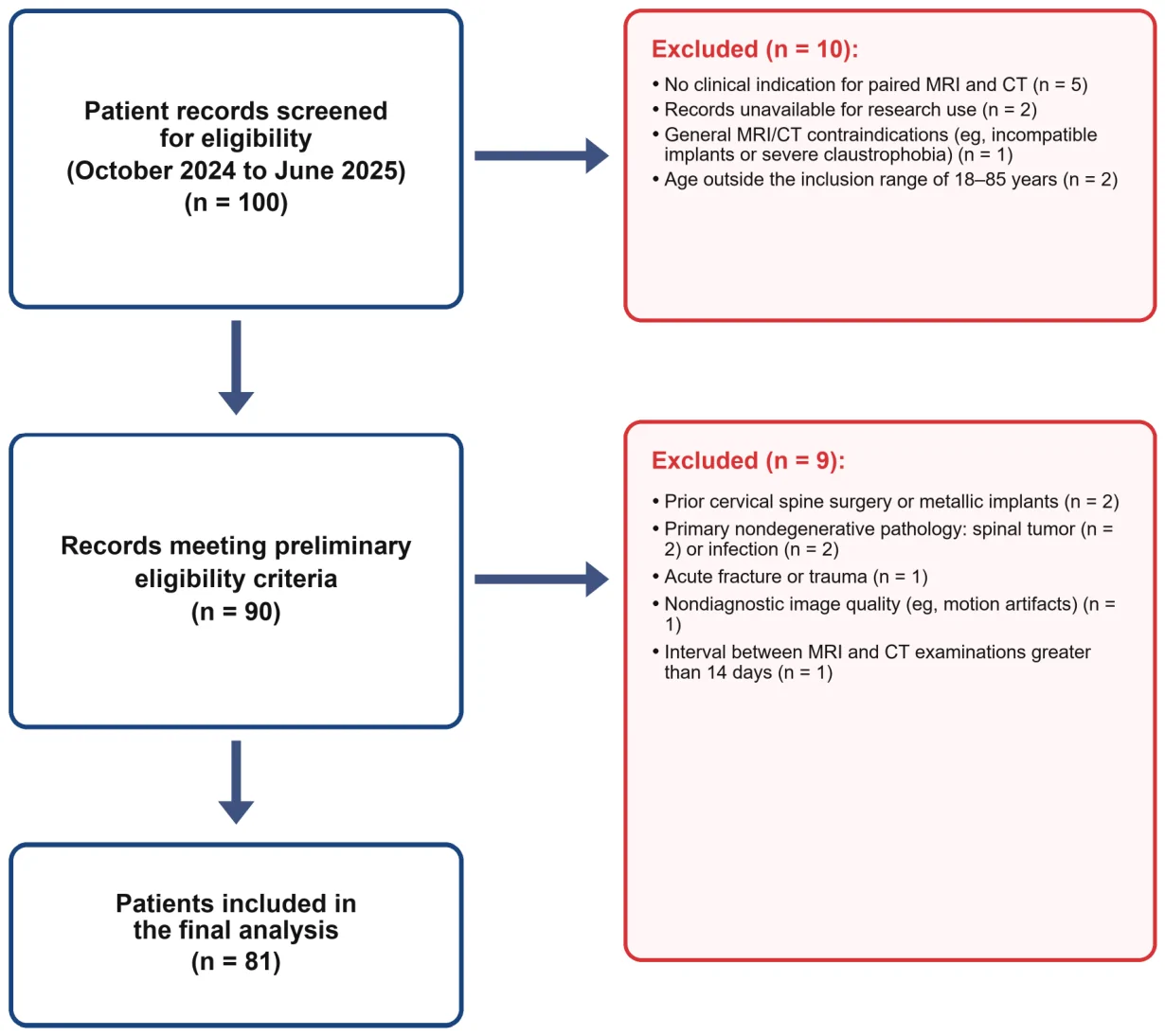
Patient selection. Of 100 consecutive patients evaluated between October 2024 and June 2025, 81 met the prespecified criteria and had MRI and CT examinations within 14 days. They provided 810 evaluable neural foramina from C2–C3 through C6–C7. CT, computed tomography; MRI, magnetic resonance imaging.

The source population comprised 100 consecutive patients evaluated for suspected cervical radiculopathy in a spine surgery outpatient clinic between October 2024 and June 2025 (Figure 1). Retrospective review of medical records and imaging identified 81 patients with analyzable paired MRI and CT who met the eligibility criteria. Exclusions were applied before image interpretation.

Prespecified inclusion criteria were age 18–85 years, clinical features consistent with cervical radiculopathy, and clinically indicated cervical MRI and CT within 14 days. Exclusion criteria were no indication for paired imaging, unavailable research-use records, MRI or CT contraindications, age outside the specified range, prior cervical surgery or metallic implants, tumour, infection, trauma, severe systemic illness, nondiagnostic image quality, or an imaging interval exceeding 14 days.

### 2.2. CT Protocol

CT was performed with a 256-detector-row scanner (Revolution CT; GE Healthcare, Chicago, IL, USA) using automatic tube current modulation (120 kVp; 100–400 mAs; pitch, 0.992; rotation time, 0.5 s). Imaging covered C2–T1. Images were reconstructed at a section thickness of 1.0 mm with bone and soft-tissue kernels. Bone-window images were used for the CT reference assessment.

### 2.3. MRI Protocol

MRI was performed with a 3.0-T system (SIGNA Premier; GE Healthcare) and a dedicated 21-channel phased-array cervical spine coil. Imaging covered C2–T1 without intravenous contrast.

#### 2.3.1. Conventional MRI Sequence

The conventional protocol included sagittal T1-weighted and T2-weighted fast spin-echo (FSE) sequences and an axial T2-weighted FSE sequence, followed by three-dimensional ZTE imaging. For sagittal T1-weighted and T2-weighted FSE imaging, the respective repetition time/echo time values were 805/7.4 ms and 3000/90 ms. Both sagittal sequences used a section thickness of 3.0 mm and an interslice gap of 0.3 mm. Conventional MRI acquisition time was 11 min 41 s.

#### 2.3.2. ZTE Sequence

ZTE imaging is designed to sample rapidly decaying signals from short-T2 tissues, providing bone-sensitive contrast that is complementary to conventional soft-tissue MRI [3,4].

Three-dimensional ZTE imaging (Silenz) was acquired with a repetition time of 859.54 ms, nominal echo time of 0 ms, flip angle of 2°, field of view of 180 × 180 mm^2^, in-plane acquisition matrix of 188 × 188, nominal acquired voxel size of approximately 1.0 × 1.0 × 1.0 mm^3^, receiver bandwidth of 50 Hz/pixel, and four signal averages. No acquisition acceleration was used. The ZTE acquisition time was 3 min 30 s.

Reconstruction used conventional filtering and interpolation. Grayscale inversion provided CT-like bone contrast without additional bone-enhancement processing. Reported voxel dimensions describe nominal acquisition resolution, not interpolated reconstruction.

### 2.4. Image Analysis

Image quality, ZTE–CT morphometric agreement, and reader-specific diagnostic performance were assessed with RadiAnt DICOM Viewer (version 4.2.1; Medixant, Poznan, Poland).

Before study readings, two board-certified musculoskeletal radiologists with 17 and 9 years of experience completed calibration using 20 cases outside the study sample. They independently scored CT, conventional MRI, ZTE, and combined MRI + ZTE datasets, then discussed disagreements and the scoring criteria. Calibration covered the five-point image-quality scale and the four-category stenosis scale (Supplementary Figure S1). The calibration was performed to standardise interpretation before independent study readings [17].

#### 2.4.1. Qualitative Image Assessment

The two radiologists independently evaluated axial ZTE images while blinded to clinical information and other imaging findings. Each of the 81 examinations received one score per domain for cortical margin definition, artefacts, intraforaminal space delineation, and overall diagnostic readability. Scores used a 5-point Likert scale (1, nondiagnostic or poorest quality; 5, best quality). Each reader repeated the assessment after 2 weeks.

#### 2.4.2. Quantitative Image and Morphometric Analysis

Two radiologists independently measured ZTE and CT images to assess morphometric agreement and interreader reliability.

Oblique sagittal assessment is useful for the obliquely oriented cervical neural foramina [18]. Bilateral anteroposterior (AP) and superoinferior (SI) diameters from C2-C3 through C6-C7 were measured on reconstructed oblique sagittal ZTE and CT images perpendicular to each foramen’s long axis. CT was the reference modality.

Global cervical alignment was measured as the Cobb angle between the inferior endplates of C2 and C7 on sagittal CT and ZTE images. This assessed inter-examination alignment differences but could not exclude local positional effects on foraminal measurements.

#### 2.4.3. Diagnostic Performance Assessment

The same two radiologists independently reviewed anonymised conventional MRI and MRI + ZTE datasets in separate sessions at least 4 weeks apart. They were blinded to recorded CT reference results, each other’s interpretations, and clinical information other than suspected cervical radiculopathy.

##### Reference Standard Definition

CT examinations were performed independently of MRI and ZTE findings. The same two radiologists who interpreted MRI and MRI + ZTE established the CT consensus reference. They were blinded to clinical information other than suspected cervical radiculopathy. Discrepant CT grades were resolved by consensus. Only final consensus grades were retained; individual pre-consensus grades were not preserved, so initial CT interreader agreement could not be estimated.

##### Stenosis Grading Scale

The same study-specific 4-point qualitative scale was applied to CT, conventional MRI, and MRI + ZTE: grade 0, normal; grade 1, mild; grade 2, moderate; and grade 3, severe stenosis (Supplementary Figure S1). Grading incorporated osseous narrowing, nerve-root morphology, and surrounding fat appearances. The <25%, 25–50%, and >50% boundaries were visual anchors; narrowing percentages were not calculated. These boundaries have precedent in cervical ZTE research [12], but this composite scale has not been independently validated against symptomatic nerve-root compromise. The term “clinically significant” in Figure S1 identifies the operational imaging threshold; clinical significance was not validated against symptoms or treatment need. Consensus CT grades 2 and 3 defined a positive reference result, and index-test grade ≥2 defined a positive MRI result.

##### Blinded Reading Sessions

Protocol order was counterbalanced: Reader 1 assessed MRI alone first and MRI + ZTE second; Reader 2 used the reverse order. For MRI alone, readers assessed sagittal T1-weighted, sagittal T2-weighted, and axial T2-weighted images. For MRI + ZTE, readers integrated conventional MRI and corresponding coregistered ZTE images to assign a grade to each foramen.

### 2.5. Sample Size and Precision Considerations

Sample size was determined by consecutive eligible examinations available during the prespecified study period, without an a priori recruitment target. Patient-clustered confidence intervals (CIs) described the precision of reader-specific paired protocol differences.

### 2.6. Statistical Analysis

Analyses used EmpowerStats (X&Y Solutions, Shenzhen, China), SPSS (version 22.0; IBM, Armonk, NY, USA), and Python (version 3.12; Python Software Foundation, Wilmington, DE, USA). Continuous variables are summarised as mean ± standard deviation or median (interquartile range), and categorical variables as counts and percentages. Reader agreement was assessed with weighted κ; image-quality reliability used linear weights. Repeat quality scores were compared using paired Wilcoxon signed-rank tests. Intermodality agreement was assessed with intraclass correlation coefficients (ICCs) and Bland–Altman analysis [17,19]. Lin’s concordance correlation coefficient was also reported for cervical alignment [20].

Diagnostic performance was evaluated separately for each reader. Sensitivity, specificity, accuracy, positive predictive value, and negative predictive value were reported with descriptive Wilson score 95% CIs [21]. Areas under the receiver operating characteristic curve (AUCs) had DeLong CIs [22]. These unclustered intervals describe observed foramen-level performance and do not account for within-patient dependence.

The principal analysis used paired patient-clustered bootstrap comparisons of MRI + ZTE with MRI [23,24]. For each of 5000 replicates, 81 patients were sampled with replacement; all 10 foramina and paired ratings from each sampled patient were retained. Reader-specific differences in sensitivity, specificity, accuracy, and AUC were recalculated. These comparisons estimate patient-sampling uncertainty for the two study readers but do not capture variability across readers. Unclustered McNemar and DeLong *p* values were supportive only. Tests were two-sided, with *p* < 0.05 indicating statistical significance.

A post hoc exploratory analysis stratified detection sensitivity by consensus CT grade 2 or 3, with index-test grade ≥2 retained as the positivity threshold. Reader-specific paired differences (MRI + ZTE minus MRI) and nominal 95% percentile CIs were estimated with 5000 patient-clustered bootstrap resamples, retaining all foramina and paired readings within each sampled patient. These analyses were not adjusted for multiple comparisons.

## 3. Results

### 3.1. Patient Flow and Baseline Characteristics

The study included 81 patients (mean age, 48.9 ± 13.6 years; 55 women; Figure 1 and Table 1). All 810 foramina from C2–C3 through C6–C7 had evaluable index-test and reference-standard results, with no missing or indeterminate assessments. Consensus CT grades were 0 in 412 foramina (50.9%), 1 in 248 (30.6%), 2 in 87 (10.7%), and 3 in 63 (7.8%).

**Figure 2:**
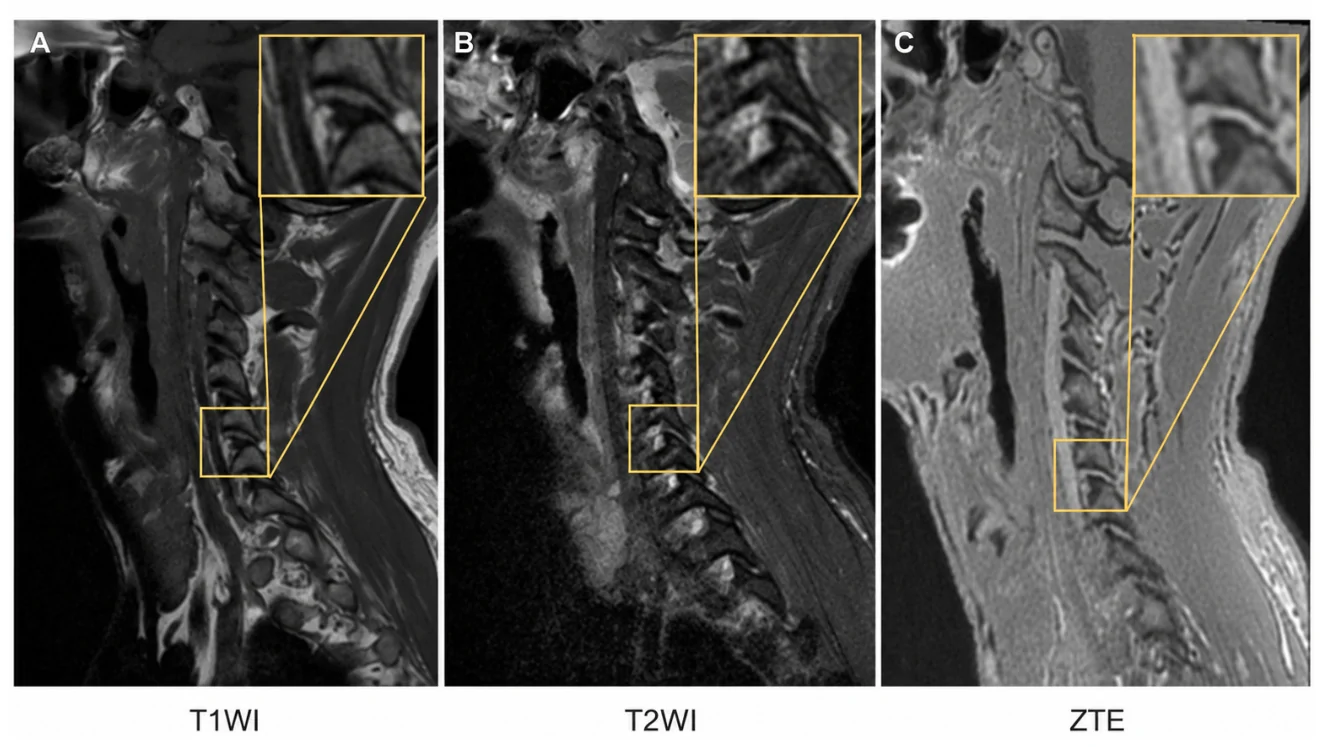
Left C5–C6 Foraminal Stenosis in a 59-year-old Man. (**A**) T1-weighted, (**B**) T2-weighted, and (**C**) ZTE images. Boxes locate the target foramen; square insets in the upper-right corner of each panel show cropped enlargements. Paired connector lines identify the source regions. The lesion was classified as grade 2 on the study’s CT-referenced composite scale. ZTE, zero-echo-time imaging.

At the grade ≥2 threshold, 150 foramina (18.5%) were CT-positive and 660 were CT-negative. These consensus classifications were the reference for comparing conventional MRI with MRI + ZTE. Figure 2 shows a representative CT grade 2 case.

### 3.2. Secondary Descriptive Findings: ZTE Image Quality and Morphometric Agreement with CT

#### 3.2.1. Subjective Image Quality and Reader Reliability

The image-quality analysis included 81 examinations. Median scores were 5 in all domains, and initial mean scores ranged from 4.84 ± 0.37 to 4.94 ± 0.29. Most scores were 4 or 5, indicating a ceiling effect.

Intrareader linear weighted κ ranged from 0.54 to 1.00 for Reader 1 and from 0.85 to 0.95 for Reader 2 (Table 2; Supplementary Figure S2). Interreader κ was 0.95 (95% CI, 0.81–1.00) for osseous margin definition, 0.73 (0.43–0.93) for artefacts, 0.54 (−0.02 to 0.83) for overall diagnostic readability, and 0.45 (0.16–0.70) for intraforaminal space delineation.

For repeat scoring after 2 weeks, paired Wilcoxon signed-rank tests did not detect differences in any image-quality domain (all *p* > 0.17). These tests did not establish equivalence between the initial and repeat assessments.

#### 3.2.2. Cervical Alignment Consistency

Global cervical alignment was compared between ZTE and CT to provide context for morphometric agreement. In 81 examinations, mean C2–C7 Cobb angles were 11.94 ± 8.41° on ZTE and 12.45 ± 8.45° on CT.

Mean bias (ZTE − CT) was −0.51° (95% CI, −1.02° to 0.01°), and the mean absolute difference was 2.13 ± 1.17°. The 95% limits of agreement were −5.18° to 4.16°, with 1 of 81 paired measurements outside these limits (Supplementary Figure S3). The absolute-agreement intraclass correlation coefficient (ICC) and Lin’s concordance correlation coefficient were both 0.96 (95% bootstrap CI, 0.94–0.97).

#### 3.2.3. Morphometric Agreement with CT and Measurement Reliability

The secondary morphometric analysis described ZTE–CT agreement for AP and SI foraminal diameters in 810 foramina from 81 patients. Measurements were averaged across the two readers for each foramen within each modality.

**Figure 3:**
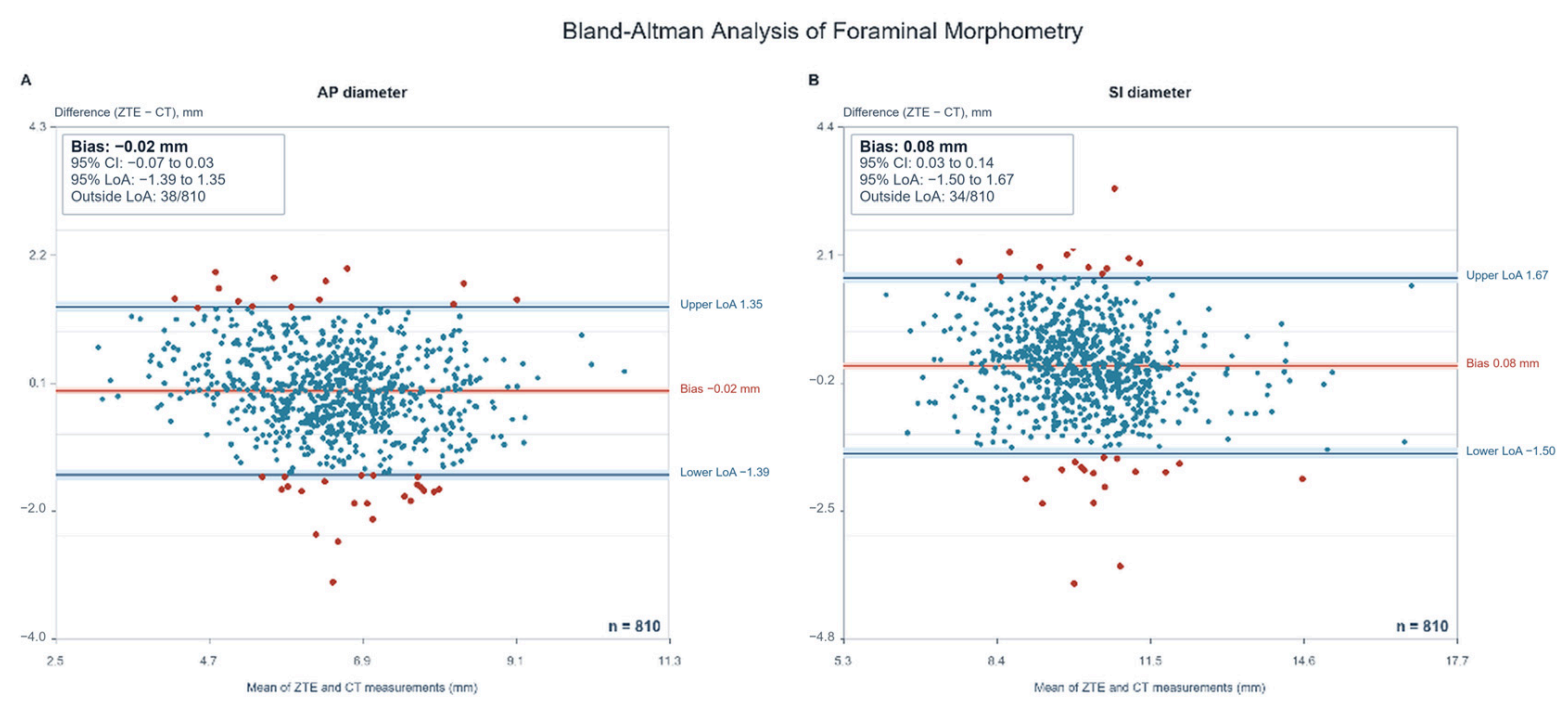
Bland–Altman Analysis of ZTE and CT Foraminal Measurements. Paired (**A**) AP and (**B**) SI diameters from 810 foramina in 81 patients were pooled across bilateral C2–C3 through C6–C7 sites. Each point represents one foramen, with measurements averaged across the two readers within each modality. The red line shows mean bias (ZTE − CT), and blue dashed lines show the 95% limits of agreement. Shading denotes 95% CIs for the bias and limits. Red points fall outside these statistical limits, which are not prespecified clinical acceptance limits. AP, anteroposterior; SI, superoinferior; ZTE, zero-echo time; CT, computed tomography; CI, confidence interval.

For AP diameter, mean bias (ZTE − CT) was −0.02 mm (95% CI, −0.07 to 0.03 mm), with 95% limits of agreement of −1.39 to 1.35 mm. Of the 810 paired measurements, 38 (4.7%) were outside these limits (Figure 3).

For SI diameter, mean bias was 0.08 mm (95% CI, 0.03–0.14 mm), with 95% limits of agreement of −1.50 to 1.67 mm. Of the 810 paired measurements, 34 (4.2%) were outside these limits. Level-specific measurements and interreader ICCs within each modality are reported in Supplementary Table S1. No clinically acceptable difference was prespecified; these findings are descriptive agreement estimates rather than evidence of measurement interchangeability.

### 3.3. CT-Referenced Diagnostic Performance and Interreader Agreement

Conventional MRI and MRI + ZTE were compared separately for each reader for detection of consensus CT grade ≥2 foraminal stenosis. Index-test grade ≥2 defined a positive result.

Among 150 CT-positive foramina, sensitivity increased from 68.7% (103/150) with conventional MRI to 87.3% (131/150) with MRI + ZTE for Reader 1, and from 17.3% (26/150) to 35.3% (53/150) for Reader 2. Among 660 CT-negative foramina, specificity increased from 90.5% (597/660) to 96.8% (639/660) and from 96.7% (638/660) to 97.6% (644/660), respectively. Accuracy increased from 86.4% to 95.1% and from 82.0% to 86.0%, respectively. AUC increased from 0.852 to 0.967 for Reader 1 and from 0.685 to 0.807 for Reader 2 (Table 3).

False negatives decreased from 47 to 19 for Reader 1 and from 124 to 97 for Reader 2. False positives decreased from 63 to 21 and from 22 to 16, respectively (Table 3; Supplementary Figure S4). Positive and negative predictive values and descriptive, unclustered CIs for the absolute performance estimates are provided in Table 3.

#### 3.3.1. Patient-Level Clustered Bootstrap Analysis

Paired patient-clustered comparisons accounted for dependence among foramina within each patient. Sensitivity differences were 18.7 percentage points (95% clustered bootstrap CI, 10.6–27.1) for Reader 1 and 18.0 percentage points (6.6–30.3) for Reader 2. Corresponding AUC differences were 0.115 (0.076–0.164) and 0.121 (0.061–0.184), respectively (Table 4).

Specificity differences were 6.4 percentage points (95% clustered bootstrap CI, 3.2–9.8) for Reader 1 and 0.9 percentage points (−1.2 to 3.0) for Reader 2. The interval for Reader 2 included zero. Accuracy differences were 8.6 percentage points (5.9–11.6) and 4.1 percentage points (1.7–6.7), respectively.

#### 3.3.2. Interreader Agreement in Stenosis Grading

For four-category stenosis grades, interreader weighted κ increased numerically from 0.31 with conventional MRI to 0.40 with MRI + ZTE. For binary grade ≥2 classification, κ increased numerically from 0.25 to 0.41.

#### 3.3.3. Exploratory Analysis Stratified by CT Grade

For CT grade 2, sensitivity increased from 55.2% (48/87) to 78.2% (68/87) for Reader 1 and from 18.4% (16/87) to 27.6% (24/87) for Reader 2. Paired differences were 23.0 (nominal 95% CI, 12.4–33.7) and 9.2 (−5.1 to 24.8) percentage points, respectively. For CT grade 3, sensitivity increased from 87.3% (55/63) to 100.0% (63/63) for Reader 1 and from 15.9% (10/63) to 46.0% (29/63) for Reader 2. Reader 2 still missed 63 grade 2 and 34 grade 3 foramina with MRI + ZTE (Supplementary Table S2; Figure S5).

## 4. Discussion

Adding ZTE improved detection of CT-defined grade ≥2 foraminal stenosis in this retrospective sample. Patient-clustered comparisons supported improvements in sensitivity and AUC for both readers, but absolute performance remained markedly reader-dependent. Interreader agreement increased numerically; differences in κ were not formally tested. These findings support the value of additional bone-sensitive information while showing that diagnostic interpretation remained inconsistent across readers.

Argentieri et al. reported ZTE–CT grading agreement of weighted κ = 0.72 using a six-category scale in 34 patients. In a 10-patient subset, interreader weighted κ was 0.71 for ZTE and 0.75 for CT [8]. These values cannot be compared directly with our four-category interreader κ of 0.40 for MRI + ZTE because the scales, readers, and protocols differed. She et al. reported CT–FRACTURE weighted κ values of 0.689–0.715 with a different four-grade scale [25]. Those values describe intermodality agreement, not interreader agreement [13]. CT grading also depends on readers and reconstruction, as shown by differences between axial and splayed CT reformations [26].

Engel et al. reported improved recognition of osseous cervical foraminal stenosis with susceptibility-weighted MRI compared with conventional sequences [27]. Their scale incorporated perineural fat and nerve-root morphology. Our composite scale also considered neural and fat appearances, but its category definitions and sequence implementation differed, preventing direct numerical comparison.

Recent cervical bone-MRI studies have examined related but different diagnostic targets. In 137 prospectively studied patients, Wang et al. reported better detection of osseous abnormalities and greater diagnostic confidence with FRACTURE than with routine MRI. They assessed morphological foraminal narrowing at eight sites; we used a CT grade ≥ 2 threshold at ten sites [11]. Differences in target definition, coverage, disease severity, and sequence implementation may explain variation in performance. Tran et al. assessed osseous contributions to narrowing and diagnostic confidence after adding ZTE, rather than CT-referenced accuracy [9]. In a cadaveric CT-comparison study, Deininger-Czermak et al. found that UTE and FRACTURE added osseous information and changed stenosis grades, although MRI still classified stenosis as insignificant more often than CT [10]. These findings distinguish additional bone information from reliable detection at a defined threshold.

Reconstruction methods may also contribute to between-study differences. Kaniewska et al. reported improved bone visualisation with deep learning reconstruction of ZTE, but CT comparisons included only 12 of 52 patients, and their overall structure-assessment ICCs measured a broader outcome than our stenosis-specific κ [12]. Peng et al. reported better ZTE image quality and acquisition efficiency, without validating detection of CT-defined moderate-to-severe foraminal stenosis [28]. Jardon et al. evaluated foraminal grades with deep-learning-reconstructed conventional MRI [29]. Van der Kolk et al. reported image-quality noninferiority of deep-learning-derived synthetic CT for overall bone depiction and most assessed cervical structures [30]. That synthesis-based method differs from directly acquired ZTE, and image-quality noninferiority does not establish diagnostic noninferiority. These studies suggest protocol improvements but do not show that reconstruction alone resolves reader-dependent performance.

High subjective ZTE quality did not ensure consistent interpretation. Median scores were 5 across all domains, but interreader linear weighted κ was 0.54 (95% CI, −0.02 to 0.83) for diagnostic readability and 0.45 (0.16–0.70) for intraforaminal space delineation. Ratings concentrated at 4 and 5, which restricted variation and may have influenced κ through their marginal distributions. The wide CIs indicate limited precision. These examination-level quality ratings address a different task from stenosis detection [17]. Despite calibration with 20 nonstudy cases, readers may differ in visual estimates near the 25% and 50% boundaries and in interpreting neural and fat appearances. Reader 2’s low sensitivity and high specificity may reflect a more conservative classification pattern, but the cause remains uncertain. Review of discordant cases is needed to distinguish threshold differences from image limitations. Exploratory results suggest benefit in CT grade 2 for Reader 1, while improvement for Reader 2 remained uncertain. Reader 2’s MRI + ZTE sensitivity was only 46.0% even in CT grade 3.

Jiang et al. reported higher interreader agreement with FRACTURE-based nerve/bone fusion imaging than with T2-weighted imaging, using a three-category scale based on foraminal and nerve-root widths [31]. Different category definitions and the fusion technique prevent those results from validating our four-category composite scale.

Interreader ICCs for foraminal measurements were numerically lower on ZTE than on CT. Overall AP ICCs were 0.68 (95% CI, 0.63–0.72) and 0.75 (0.71–0.78), respectively; SI ICCs were 0.71 (0.66–0.75) and 0.78 (0.75–0.81) (Supplementary Table S1). A paired ICC comparison was not performed, and overlapping CIs do not establish a difference. Nominal ZTE voxel size and CT section thickness do not imply identical effective resolution or boundary contrast. ZTE provides contrast from short-T2 proton signals and processing, whereas CT measures X-ray attenuation; acquisition and reconstruction affect bone conspicuity [3,5]. Edge definition, partial-volume effects, and oblique-plane selection may contribute to measurement variability, but their effects were not quantified.

Small mean ZTE–CT biases coexisted with individual measurement differences. Limits of agreement extended approximately 1.5 mm on either side of the mean, and SI measurements had a small positive bias. No clinically acceptable difference was prespecified, so interchangeability cannot be inferred [19]. The analysis used reader-averaged measurements and does not establish agreement for an individual reader. Agreement in global C2–C7 alignment does not exclude local positional or plane-selection effects.

Pan et al. reported ZTE–CT agreement for several cervical dimensions, with lower agreement for minimum measurements, particularly pedicle dimensions [32]. They analysed patient-level summaries in 38 surgical patients; our analysis used individual foramina and reader-averaged measurements. Morbée et al. demonstrated equivalence of selected lumbar synthetic-CT measurements within prespecified margins [33]. Despite differences in anatomy and synthesis method, that design illustrates why a small mean bias alone does not establish equivalence. Our study did not prespecify acceptable measurement differences.

Evidence from other diagnostic tasks cannot be transferred directly to degenerative foraminal stenosis. Lecouvet et al. reported high ZTE and black-bone MRI accuracy for myeloma-related osteolysis, with more ZTE false negatives in the spine [34]. Fischer et al. reported high fracture sensitivity and strong intermodality agreement in injury classification with MRI-derived synthetic CT in a prospective multicentre study [35]. These studies used different disease targets, and the latter used synthesis rather than directly acquired ZTE.

The additional 3 min 30 s of ZTE acquisition provided complementary bone-sensitive information, but Reader 2 still missed 97 of 150 CT-positive foramina. A negative MRI + ZTE interpretation therefore cannot reliably exclude CT-defined grade ≥2 stenosis. These findings support further evaluation of ZTE as an add-on sequence, but they do not validate reduced CT use or improved treatment outcomes [1,6].

This study has limitations. The single-centre, single-vendor setting and selection of patients who required both MRI and CT limit generalizability and may introduce spectrum and selection bias. Sample size was fixed, and two readers cannot characterise a broader reader population or reader-by-protocol interactions [17,24]. Sex-specific differences were not evaluated. Anonymisation, counterbalanced reading order, and a minimum 4-week interval reduced the likelihood of recall but could not eliminate memory effects. The same readers established the CT consensus reference, creating potential observer bias. Because pre-consensus grades were not preserved, CT grading reliability could not be estimated or compared with MRI grading reliability. The study-specific scale combined narrowing with neural and fat appearances; its grade ≥ 2 threshold has not been validated against symptoms or operative findings. CT is also a limited reference for the neural and perineural features in the scale. CT consensus is an anatomical classification, not proof of symptomatic nerve-root involvement or surgical truth, and it cannot assess dynamic narrowing. Image-quality and morphometric analyses were secondary and descriptive. CT-grade-stratified analyses were post hoc and unadjusted for multiple comparisons. Prospective multicentre studies should include more readers, standardised calibration, review of diagnostic disagreements, and clinical or operative correlation to assess effects on CT use, management, and patient outcomes.

## 5. Conclusions

Adding ZTE improved detection of CT-defined grade ≥ 2 cervical foraminal stenosis in this study, although sensitivity remained low for one reader. The findings support further validation of ZTE as a bone-sensitive adjunct. Prospective multicentre studies with standardised acquisition and alignment protocols and larger reader samples should determine effects on CT use, clinical management, and patient outcomes.

## Figures and Tables

**Table 1:** Baseline Demographic, Clinical, and Imaging Characteristics.

Characteristic	Value (N = 81 Patients)
Age (years)	48.9 ± 13.6
Male	26 (32.1%)
Female	55 (67.9%)
Duration of symptoms (months) *	6.5 (3.0–14.0)
Primary symptom side *	
Left	32 (39.5%)
Right	37 (45.7%)
Bilateral	12 (14.8%)
Predominant radicular symptoms *	
Radicular pain/neuropathic pain	49 (60.5%)
Sensory deficit/numbness	20 (24.7%)
Motor weakness	12 (14.8%)
Imaging characteristics (reference CT)	(N = 810 foramina)
Evaluated levels	C2–C3 to C6–C7
Grade 0 (normal)	412 (50.9%)
Grade 1 (mild)	248 (30.6%)
Grade 2 (moderate)	87 (10.7%)
Grade 3 (severe)	63 (7.8%)
Moderate-to-severe stenosis (Grade ≥2)	150 (18.5%)

Data are patient or foramen counts (percentages), mean ± standard deviation, or median (interquartile range). * Clinical data were extracted from the institutional database. CT, computed tomography.

**Table 2:** Subjective ZTE Image Quality and Reader Reliability.

Domain	Reader 1 Score	Reader 2 Score	Intrareader κ: Reader 1	Intrareader κ: Reader 2	Interreader κ
Osseous foraminal margin definition	4.85 ± 0.39	4.86 ± 0.38	0.85 (0.66–1.00)	0.95 (0.79–1.00)	0.95 (0.81–1.00)
Absence/control of image artefacts	4.89 ± 0.35	4.85 ± 0.39	0.54 (0.16–0.82)	0.85 (0.63–1.00)	0.73 (0.43–0.93)
Overall diagnostic readability	4.89 ± 0.35	4.94 ± 0.29	0.94 (0.78–1.00)	0.90 (0.49–1.00)	0.54 (−0.02 to 0.83)
Intraforaminal space delineation	4.84 ± 0.37	4.84 ± 0.37	1.00 (1.00–1.00)	0.87 (0.72–1.00)	0.45 (0.16–0.70)

Each reader scored 81 patient examinations and repeated scoring after 2 weeks. Image quality was graded on a 5-point Likert scale (1: nondiagnostic; 5: excellent). Scores are expressed as mean ± SD from the initial reader assessment. Reader reliability was evaluated using linear weighted κ statistics with bootstrap 95% CIs. CI, confidence interval; SD, standard deviation; ZTE, zero-echo time.

**Table 3:** Reader-specific detection of CT-defined Grade ≥2 Foraminal Stenosis.

Metric	Reader 1: MRI	Reader 1: MRI + ZTE	Reader 2: MRI	Reader 2: MRI + ZTE
CT-positive TP/FN	103/47	131/19	26/124	53/97
CT-negative FP/TN	63/597	21/639	22/638	16/644
Sensitivity, % (95% CI)	68.7 (60.9–75.5)	87.3 (81.1–91.7)	17.3 (12.1–24.2)	35.3 (28.1–43.3)
Specificity, % (95% CI)	90.5 (88.0–92.5)	96.8 (95.2–97.9)	96.7 (95.0–97.8)	97.6 (96.1–98.5)
Accuracy, % (95% CI)	86.4 (83.9–88.6)	95.1 (93.3–96.4)	82.0 (79.2–84.5)	86.0 (83.5–88.3)
PPV, % (95% CI)	62.0 (54.5–69.1)	86.2 (79.8–90.8)	54.2 (40.3–67.4)	76.8 (65.6–85.2)
NPV, % (95% CI)	92.7 (90.4–94.5)	97.1 (95.5–98.1)	83.7 (80.9–86.2)	86.9 (84.3–89.1)
AUC (95% CI)	0.852 (0.817–0.887)	0.967 (0.956–0.979)	0.685 (0.641–0.729)	0.807 (0.768–0.845)

CT grade ≥2 defined positive stenosis. Intervals for absolute proportions use Wilson scores and those for AUC use DeLong; these are descriptive, unclustered intervals. The principal clustered analysis is in Table 4. Supportive unclustered *p* values for sensitivity, specificity, accuracy, and AUC were all <0.001 for Reader 1; for Reader 2, they were <0.001, 0.377, <0.001, and <0.001, respectively. TP/FN, true positive/false negative; FP/TN, false positive/true negative; PPV/NPV, positive/negative predictive value; CI, confidence interval; AUC, area under the receiver operating characteristic curve; CT, computed tomography; MRI, magnetic resonance imaging; ZTE, zero-echo-time imaging.

**Table 4:** Patient-Clustered Bootstrap Differences for MRI + ZTE versus MRI.

Reader	Metric	Difference	95% Clustered Bootstrap CI
Reader 1	Sensitivity	18.7 percentage points	10.6–27.1
Reader 1	Specificity	6.4 percentage points	3.2–9.8
Reader 1	Accuracy	8.6 percentage points	5.9–11.6
Reader 1	AUC	0.115	0.076–0.164
Reader 2	Sensitivity	18.0 percentage points	6.6–30.3
Reader 2	Specificity	0.9 percentage points	−1.2 to 3.0
Reader 2	Accuracy	4.1 percentage points	1.7–6.7
Reader 2	AUC	0.121	0.061–0.184

Values are reader-specific paired differences between MRI + ZTE and conventional MRI. For each of 5000 patient-level clustered bootstrap replicates, patients were sampled with replacement and all 10 foramina and paired protocol ratings from each sampled patient were retained. The patient, not the individual foramen, was the resampling unit. AUC, area under the receiver operating characteristic curve; CI, confidence interval; MRI, magnetic resonance imaging; ZTE, zero-echo-time.

## Data Availability

The deidentified data supporting the findings of this study are available from the corresponding author on reasonable request after publication, subject to ethics approval and privacy restrictions. The data are not publicly available because they contain protected clinical information.

## Supplementary Materials

The following supporting information can be downloaded at: https://www.mdpi.com/article/10.3390/diagnostics16213597/s1, Figure S1: Grading Scale for Osseous Cervical Neural Foraminal Stenosis. The schematic illustrates the 4-point qualitative grading system used to assess stenosis severity, ranging from Grade 0 (normal) to Grade 3 (severe), based on the degree of foraminal narrowing and its impact on the exiting nerve root. The positive threshold was grade ≥2; Figure S2: Reproducibility of subjective ZTE image-quality ratings. Heatmap cells show linear weighted κ and bootstrap-derived 95% confidence intervals for intrareader and interreader agreement in 81 examinations per reader. Readers repeated the assessment after 2 weeks. Darker shading indicates higher κ values.; Figure S3: ZTE–CT agreement in cervical alignment. Bland–Altman plot of C2–C7 Cobb angles in 81 paired examinations. The solid red line indicates mean bias (ZTE−CT, −0.51°), and the dashed blue lines indicate the 95% limits of agreement (−5.18° to 4.16°). One examination (1.2%) fell outside these limits. ZTE, zero echo time; CT, computed tomography; MRI, magnetic resonance imaging.; Figure S4: Diagnostic classification counts. Consensus CT grade ≥2 was the reference standard. (A) Read-er-specific 2×2 matrices for conventional MRI and MRI+ZTE. Row percentages use CT-positive or CT-negative foramina as the denominator. (B) Distributions of TP, TN, FP, and FN across readers and protocols. ZTE, zero echo time; MRI, magnetic resonance imaging; CT, computed tomography; FN, false negative; FP, false positive; TN, true negative; TP, true positive; Figure S5: Detection sensitivity stratified by CT consensus grade. (A) Sensitivity of conventional MRI and MRI+ZTE in CT grade 2 (n = 87) and grade 3 (n = 63) foramina, by reader. (B) Paired sensitivity differences (MRI+ZTE minus MRI), with nominal 95% percentile CIs from 5,000 patient-clustered bootstrap resamples. Index-test positivity remained grade ≥2 for both protocols. Analyses were post hoc and exploratory, without adjustment for multiple comparisons. CI, confidence interval; CT, computed tomography; MRI, magnetic resonance imaging; ZTE, zero-echo-time imaging.; Table S1: Foraminal dimensions and interreader reliability: (A) AP diameter (mm), (B) SI diameter (mm); Table S2: Post hoc detection sensitivity stratified by consensus CT grade.
