# Supplementary material for: Incremental Diagnostic Value of Zero-Echo-Time Magnetic Resonance Imaging for Osseous Cervical Neural Foraminal Stenosis: A Retrospective Multireader Study
Source: Diagnostics (Basel). 2026 Oct 31;16(21):3597. doi: 10.3390/diagnostics16213597 (PMC13762032; doi:10.3390/diagnostics16213597)
Supplement: Supplementary file 1 [file diagnostics-16-03597-s001.zip › diagnostics-4606628-supplementary.pdf]

Supplementary Materials

Table S1. Foraminal dimensions and interreader reliability: (A) AP diameter (mm).

Parameter and level	Side	ZTE (mean ± SD)	CT (mean ± SD)	Difference (ZTE−CT, 95% CI)	ICC ZTE (95% CI)	ICC CT (95% CI)
C2–C3	Left	6.90 ± 1.09	6.95 ± 1.20	−0.05 (−0.18 to 0.08)	0.74 (0.61–0.83)	0.73 (0.63–0.81)
	Right	6.99 ± 1.07	7.08 ± 1.08	−0.10 (−0.23 to 0.04)	0.66 (0.51–0.77)	0.74 (0.64–0.82)
C3–C4	Left	6.61 ± 1.00	6.48 ± 1.18	0.13 (−0.03 to 0.28)	0.71 (0.56–0.80)	0.67 (0.44–0.84)
	Right	6.66 ± 1.01	6.66 ± 1.12	0.00 (−0.15 to 0.16)	0.55 (0.37–0.69)	0.68 (0.54–0.78)
C4–C5	Left	6.53 ± 1.03	6.52 ± 1.17	0.00 (−0.15 to 0.15)	0.70 (0.58–0.79)	0.72 (0.61–0.80)
	Right	6.45 ± 1.04	6.50 ± 1.23	−0.05 (−0.22 to 0.13)	0.61 (0.44–0.74)	0.76 (0.63–0.84)
C5–C6	Left	6.13 ± 0.92	6.10 ± 1.10	0.02 (−0.13 to 0.18)	0.58 (0.41–0.72)	0.75 (0.61–0.84)
	Right	6.08 ± 1.21	6.08 ± 1.33	0.00 (−0.17 to 0.16)	0.69 (0.53–0.80)	0.76 (0.66–0.83)
C6–C7	Left	6.28 ± 1.02	6.38 ± 1.20	−0.10 (−0.25 to 0.05)	0.64 (0.44–0.79)	0.79 (0.70–0.85)
	Right	6.30 ± 1.12	6.39 ± 1.18	−0.08 (−0.24 to 0.07)	0.70 (0.57–0.81)	0.79 (0.68–0.87)
Overall AP	Bilateral	6.49 ± 1.09	6.51 ± 1.21	−0.02 (−0.07 to 0.03)	0.68 (0.63–0.72)	0.75 (0.71–0.78)

Table S1 (continued). (B) SI diameter (mm).

Parameter and level	Side	ZTE (mean ± SD)	CT (mean ± SD)	Difference (ZTE−CT, 95% CI)	ICC ZTE (95% CI)	ICC CT (95% CI)
C2–C3	Left	11.07 ± 1.23	11.08 ± 1.21	−0.01 (−0.19 to 0.17)	0.69 (0.49–0.79)	0.63 (0.50–0.74)
	Right	10.97 ± 1.09	10.99 ± 1.29	−0.02 (−0.21 to 0.17)	0.35 (0.13–0.65)	0.73 (0.61–0.81)
C3–C4	Left	9.87 ± 1.17	9.75 ± 1.22	0.13 (−0.02 to 0.28)	0.76 (0.59–0.85)	0.63 (0.49–0.73)
	Right	10.04 ± 1.12	9.67 ± 1.23	0.37 (0.24–0.50)	0.67 (0.50–0.78)	0.78 (0.66–0.86)
C4–C5	Left	10.05 ± 1.27	9.91 ± 1.44	0.14 (−0.06 to 0.34)	0.70 (0.58–0.78)	0.82 (0.70–0.88)
	Right	10.01 ± 1.36	9.95 ± 1.52	0.06 (−0.13 to 0.24)	0.73 (0.60–0.81)	0.80 (0.66–0.87)
C5–C6	Left	9.94 ± 1.24	9.85 ± 1.26	0.08 (−0.10 to 0.26)	0.72 (0.60–0.81)	0.72 (0.61–0.80)
	Right	9.91 ± 1.21	9.78 ± 1.47	0.14 (−0.02 to 0.30)	0.63 (0.46–0.75)	0.81 (0.73–0.87)
C6–C7	Left	9.78 ± 1.45	9.93 ± 1.46	−0.15 (−0.34 to 0.04)	0.79 (0.70–0.85)	0.81 (0.71–0.87)
	Right	9.76 ± 1.23	9.68 ± 1.40	0.08 (−0.10 to 0.26)	0.74 (0.61–0.82)	0.75 (0.65–0.83)
Overall SI	Bilateral	10.14 ± 1.31	10.06 ± 1.44	0.08 (0.03–0.14)	0.71 (0.66–0.75)	0.78 (0.75–0.81)

Values are the mean ± SD or mean ZTE−CT difference (95% CI). Dimensions and differences are based on measurements averaged across the two readers. ICCs describe interreader reliability within each modality. Overall values pool both sides at C2–C3 through C6–C7. AP, anteroposterior; SI, superoinferior; CI, confidence interval; ICC, intraclass correlation coefficient; SD, standard deviation; CT, computed tomography; ZTE, zero-echo-time imaging.

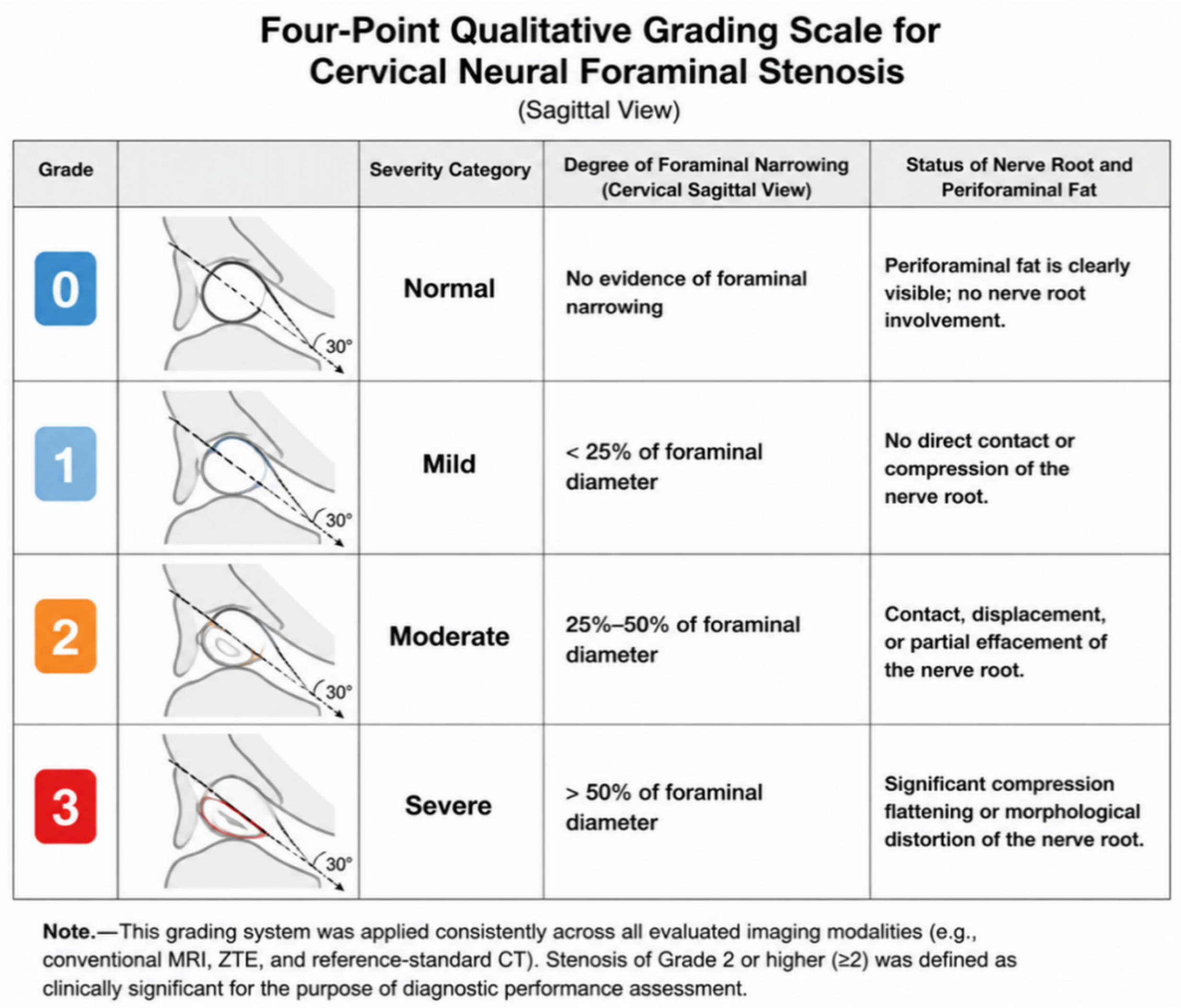

Four-Point Qualitative Grading Scale for Cervical Neural Foraminal Stenosis

(Sagittal View)

Grade		Severity Category	Degree of Foraminal Narrowing (Cervical Sagittal View)	Status of Nerve Root and Periforaminal Fat
0	30°	Normal	No evidence of foraminal narrowing	Periforaminal fat is clearly visible; no nerve root involvement.
1	30°	Mild	< 25% of foraminal diameter	No direct contact or compression of the nerve root.
2	30°	Moderate	25%–50% of foraminal diameter	Contact, displacement, or partial effacement of the nerve root.
3	30°	Severe	> 50% of foraminal diameter	Significant compression flattening or morphological distortion of the nerve root.

Note.—This grading system was applied consistently across all evaluated imaging modalities (e.g., conventional MRI, ZTE, and reference-standard CT). Stenosis of Grade 2 or higher (≥2) was defined as clinically significant for the purpose of diagnostic performance assessment.

Figure S1. Grading Scale for Osseous Cervical Neural Foraminal Stenosis. The schematic illustrates the 4-point qualitative grading system used to assess stenosis severity, ranging from Grade 0 (normal) to Grade 3 (severe), based on the degree of foraminal narrowing and its impact on the exiting nerve root. The positive threshold was grade ≥2.

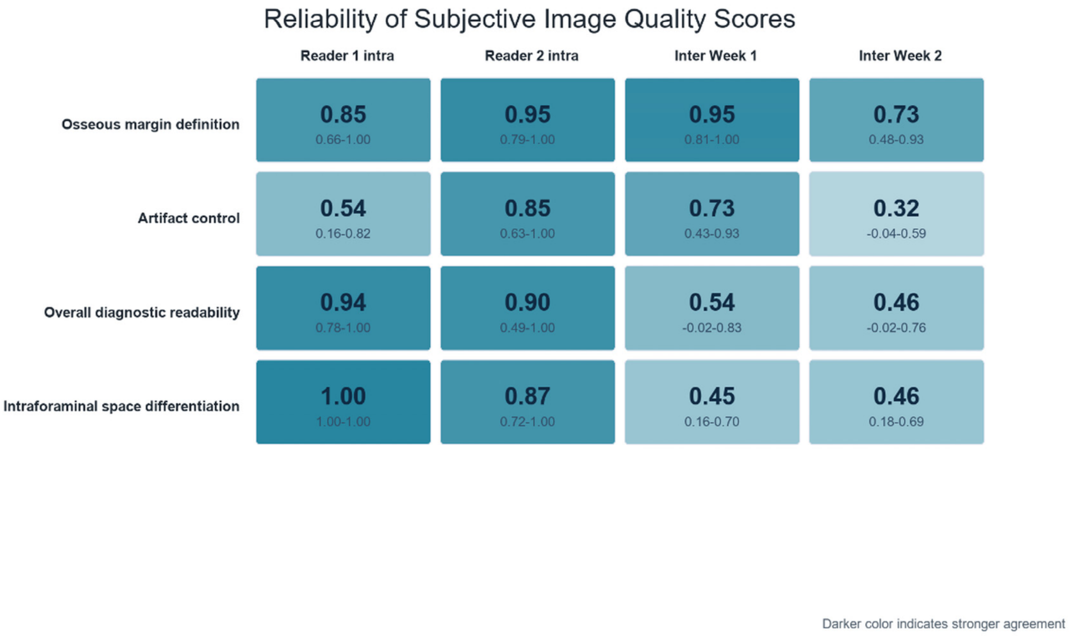

Figure S2. Reproducibility of subjective ZTE image-quality ratings. Heatmap cells show linear weighted κ and bootstrap-derived 95% confidence intervals for intrareader and interreader agreement in 81 examinations per reader. Readers repeated the assessment after 2 weeks. Darker shading indicates higher κ values.

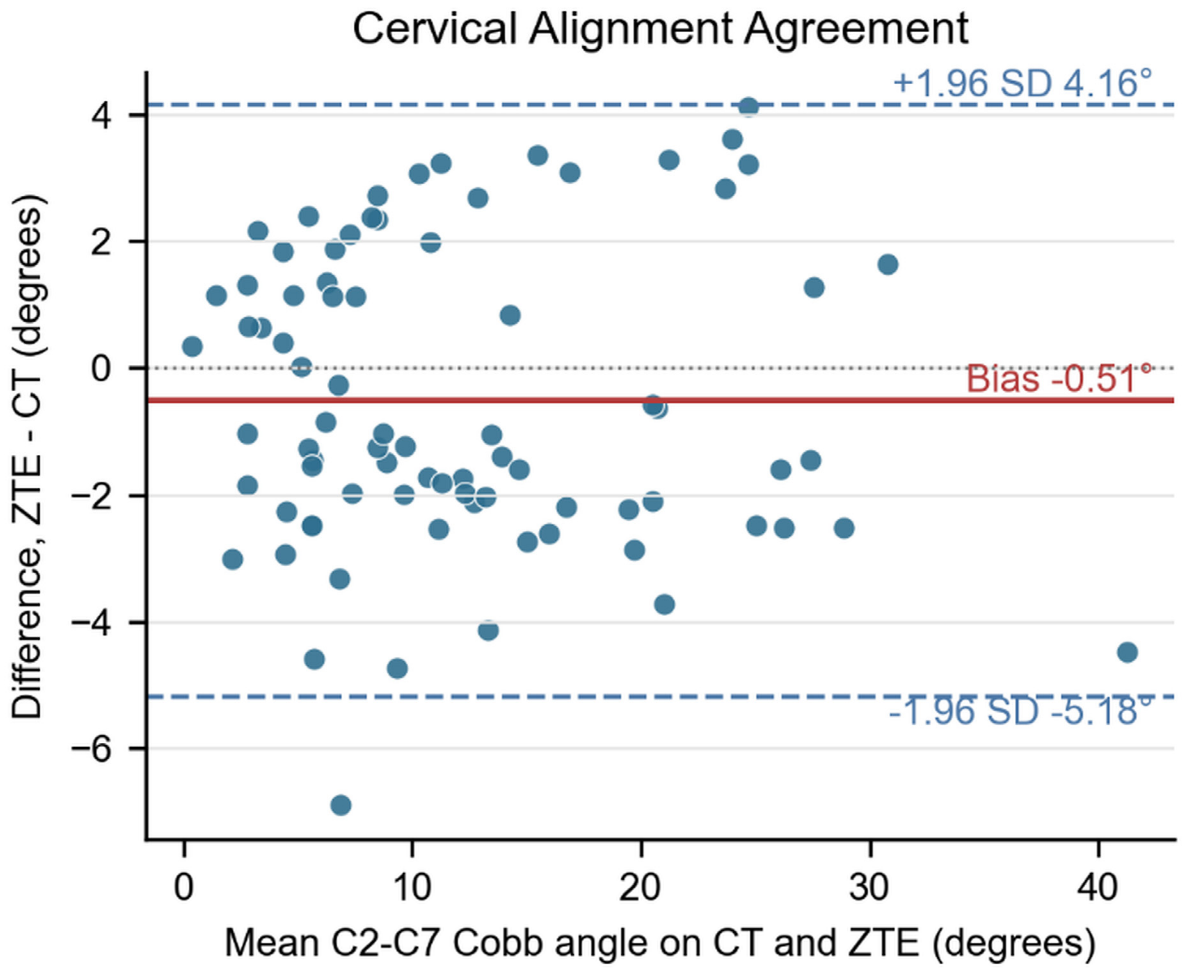

Figure S3. ZTE–CT agreement in cervical alignment. Bland–Altman plot of C2–C7 Cobb angles in 81 paired examinations. The solid red line indicates mean bias (ZTE–CT, −0.51°), and the dashed blue lines indicate the 95% limits of agreement (−5.18° to 4.16°). One examination (1.2%) fell outside these limits. ZTE, zero echo time; CT, computed tomography; MRI, magnetic resonance imaging.

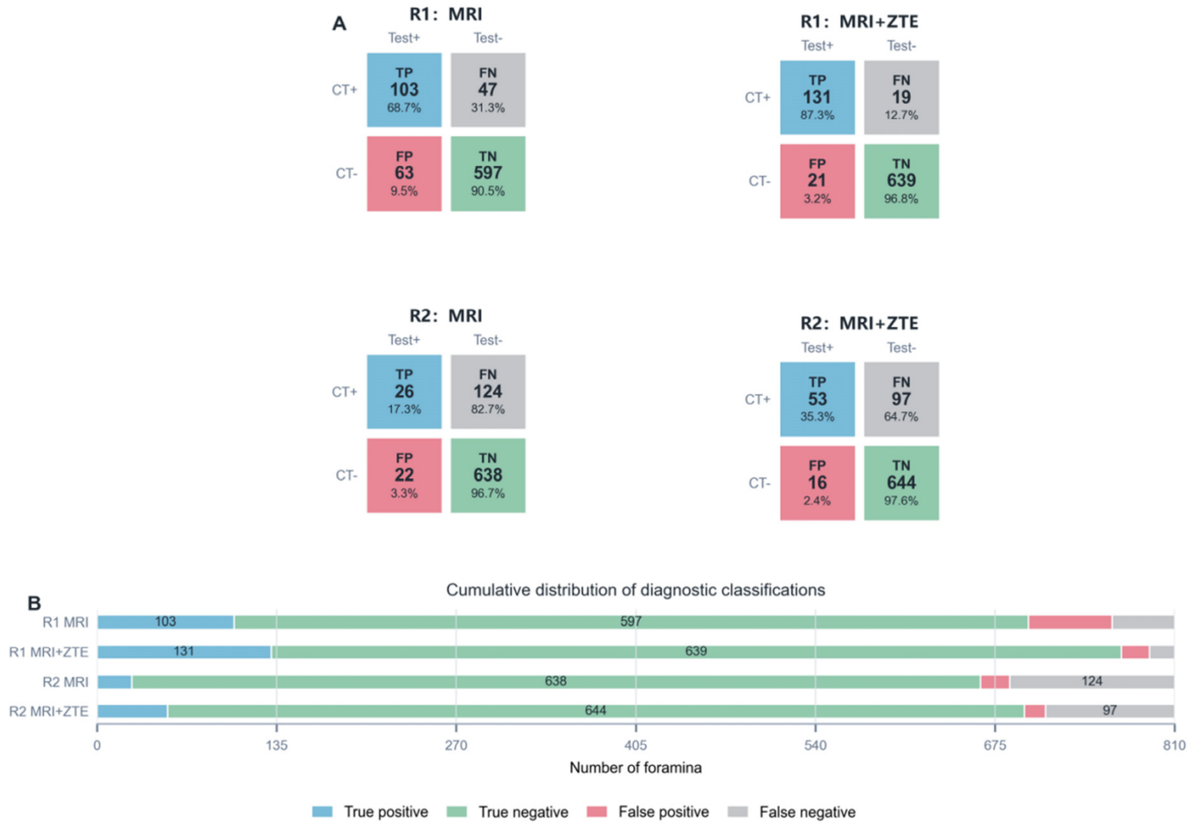

Figure S4. Diagnostic classification counts. Consensus CT grade ≥2 was the reference standard. (A) Reader-specific 2×2 matrices for conventional MRI and MRI+ZTE. Row percentages use CT-positive or CT-negative foramina as the denominator. (B) Distributions of TP, TN, FP, and FN across readers and protocols. ZTE, zero echo time; MRI, magnetic resonance imaging; CT, computed tomography; FN, false negative; FP, false positive; TN, true negative; TP, true positive.

Table S2. Post hoc detection sensitivity stratified by consensus CT grade.

CT grade	Reader	MRI sensitivity	MRI+ZTE sensitivity	Paired difference, percentage points (nominal 95% CI)
2	1	55.2% (48/87)	78.2% (68/87)	23.0 (12.4 to 33.7)
2	2	18.4% (16/87)	27.6% (24/87)	9.2 (−5.1 to 24.8)
3	1	87.3% (55/63)	100.0% (63/63)	12.7 (3.4 to 24.3)
3	2	15.9% (10/63)	46.0% (29/63)	30.2 (15.2 to 44.8)

Sensitivity is reported as a percentage (detected/CT-positive foramina). Index-test positivity was grade ≥2 for both conventional MRI and MRI+ZTE. Paired differences are MRI+ZTE minus MRI, in percentage points. Nominal 95% percentile CIs were estimated using 5,000 patient-clustered bootstrap resamples, retaining all foramina and paired readings within each sampled patient. These post hoc analyses were not adjusted for multiple comparisons. CI, confidence interval; CT, computed tomography; MRI, magnetic resonance imaging; ZTE, zero-echo-time imaging.

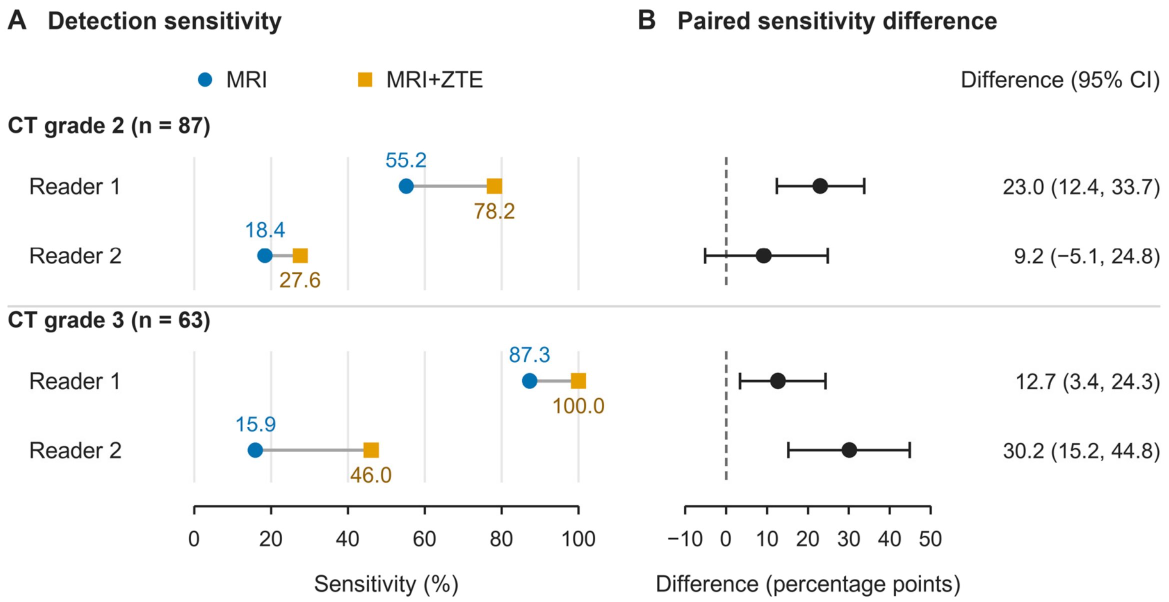

Figure S5. Detection sensitivity stratified by CT consensus grade. (A) Sensitivity of conventional MRI and MRI+ZTE in CT grade 2 (n = 87) and grade 3 (n = 63) foramina, by reader. (B) Paired sensitivity differences (MRI+ZTE minus MRI), with nominal 95% percentile CIs from 5,000 patient-clustered bootstrap resamples. Index-test positivity remained grade ≥2 for both protocols. Analyses were post hoc and exploratory, without adjustment for multiple comparisons. CI, confidence interval; CT, computed tomography; MRI, magnetic resonance imaging; ZTE, zero-echo-time imaging.
